# Synthesis, Biological Evaluation and Molecular Docking Studies of Piperidinylpiperidines and Spirochromanones Possessing Quinoline Moieties as Acetyl-CoA Carboxylase Inhibitors

**DOI:** 10.3390/molecules200916221

**Published:** 2015-09-07

**Authors:** Tonghui Huang, Jie Sun, Qianqian Wang, Jian Gao, Yi Liu

**Affiliations:** Jiangsu Key Laboratory of New Drug Research and Clinical Pharmacy, School of Pharmacy, Xuzhou Medical College, Xuzhou 221004, Jiangsu, China; E-Mails: xincheng127@sina.cn (J.S.); ruofei16888@163.com (Q.W.); gaojian.gucas@gmail.com (J.G.); cbpeliuyinew@163.com (Y.L.)

**Keywords:** acetyl-CoA carboxylase, inhibitors, piperidinylpiperidines, spirochromanones, synthesis, molecular docking

## Abstract

Acetyl-coenzyme A carboxylases (ACCs) play critical roles in the regulation of fatty acid metabolism and have been targeted for the development of drugs against obesity, diabetes and other metabolic diseases. Two series of compounds possessing quinoline moieties were designed, synthesized and evaluated for their potential to inhibit acetyl-CoA carboxylases. Most compounds showed moderate to good ACC inhibitory activities and compound **7a** possessed the most potent biological activities against ACC1 and ACC2, with IC_50_ values of 189 nM and 172 nM, respectively, comparable to the positive control. Docking simulation was performed to position compound **7a** into the active site of ACC to determine a probable binding model.

## 1. Introduction

Obesity is a chronic disease related to many other metabolic diseases such as type 2 diabetes mellitus (T2DM) and dyslipidemia [1,2]. Acetyl-coenzyme A carboxylases (ACCs) are key enzymes that catalyze the formation of malonyl coenzyme A (CoA), which contributes to the regulation of fatty acid biosynthesis and oxidation in humans and most other living organisms [3,4]. There are two isoforms of ACC in mammals identified as ACC1 and ACC2. ACC1 is a cytosolic enzyme and primarily expressed in lipogenic tissues responsible for the committed step in fatty acid biosynthesis. ACC2 is a mitochondrial enzyme that is highly expressed in oxidative tissues associated with mitochondrial fatty acid oxidation [2]. Thus, inhibition of the ACCs gives the potential for managing both long chain fatty acid biosynthesis and fatty acid oxidation, which has been regarded as a novel approach to treat obesity type 2 diabetes and other diseases associated with metabolic syndrome [5].

**Figure 1:**
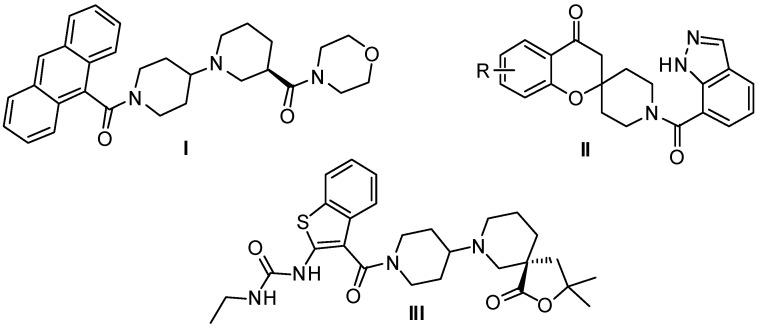
Chemical structures of representative ACC inhibitors.

Wakil and coworkers [2] have reported that the ACC2-deficient mice exhibited increased fatty acid oxidation and reduced fat accumulation in skeletal muscle and heart tissue when placed on a high fat/high calorie diet. In contrast, liver ACC1-deficient mice showed a significant reduction in fatty acid synthesis as compared to wild type mice [6]. In the past few years, several classes of small molecules that inhibit both isoforms of mammalian ACC have been reported, such as spirochromanones [7], piperidinylpiperidines [8] and benzthiazolylamides [9], examples of which are shown in Figure 1. Among them is compound CP-640186 (**I**, Figure 1) which is the first well documented example from Pfizer’s drug discovery program targeting ACCs with IC_50_ values of about 50 nM against ACC1 and ACC2 from mammalia [8,10]. In a subsequent publication, Pfizer described a series of spirochromanone ACC inhibitors (**II**, Figure 1) and discussed efforts to improve the properties [7]. Further work by Takeda focused on improving the compound of CP-640186 by replacing the anthracene ring and morpholine amide resulting in a benzthiazolylamides compound (**III**, Figure 1) [9]. Moreover, Pfizer is currently pursuing the clinical development of their clinical candidate PF-05175157 (structure not disclosed) targeting ACC in phase 2 [11].

**Figure 2:**
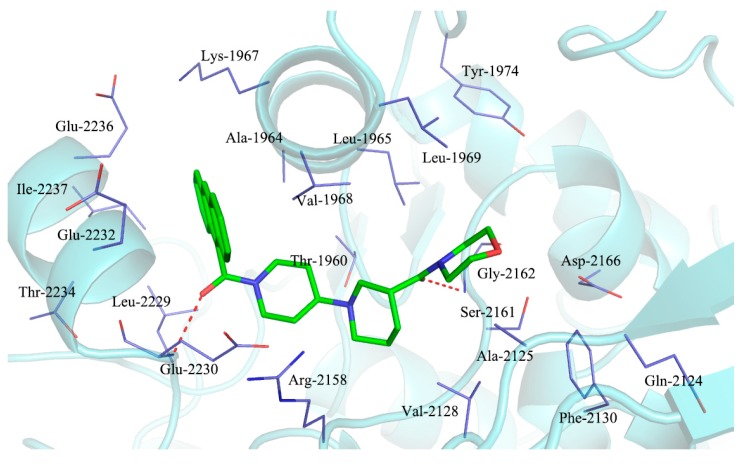
Stereographic drawing showing the binding site for CP-640186.

**Figure 3:**
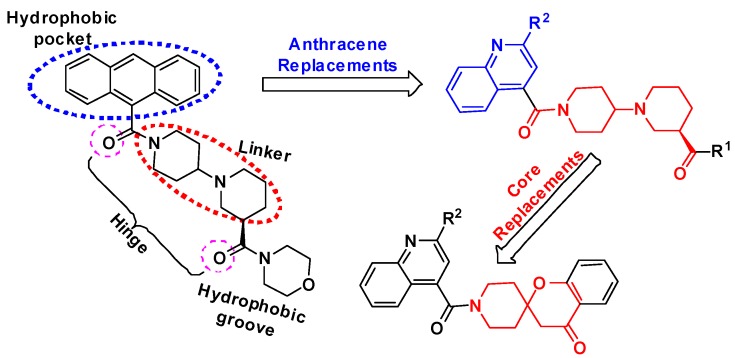
Design strategy of the target compounds.

It has been demonstrated that a spirochromanone and CP-640186 bound to the same active site at the interface of a dimer of ACC carboxyl transfer (CT) domain [12,13,14]. The shared binding pattern reveals that these two classes of inhibitors both interact with conserved residues Gly-2162 (yeast Gly-1958) and Glu-2230 (yeast Glu-2026). The crystal structure of the complex of ACC CT with CP-640186 indicates that the side chains of Glu-2230, Glu-2232, Ala-1964 and Lys-1967 are located near the anthracene ring (Figure 2) [14]. We propose that the conversion of the anthracene ring into a heterocyclic ring may enhance the interaction of ligand and the binding site, resulting in increased ACC inhibitory activities. Designs based on the anthracene regions and piperidinylpiperidine core are shown in Figure 3. To increase the structural diversity and discover more potent inhibitors of ACC, we describe herein the synthesis of novel piperidinylpiperidines and spirochromanones bearing a quinoline moiety and evaluated their enzyme inhibitory activities against ACCs. Additionally, molecular docking was carried out to investigate the possible binding mode of representative compounds using the Sybyl software.

## 2. Results and Discussion

### 2.1. Chemistry

**Scheme 1:**
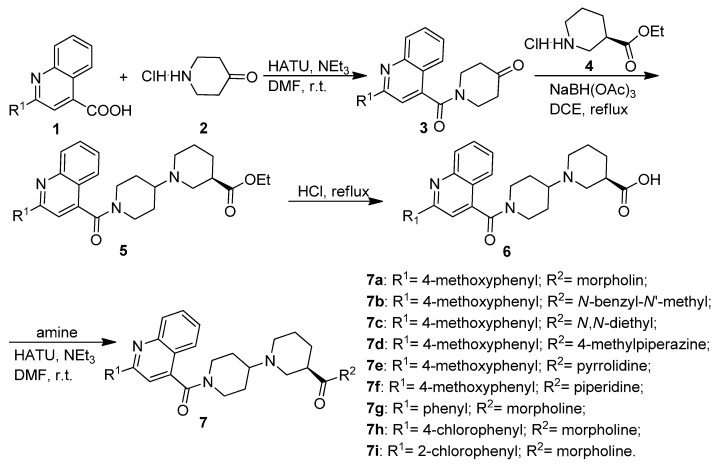
Synthesis of piperidinylpiperidines.

**Scheme 2:**
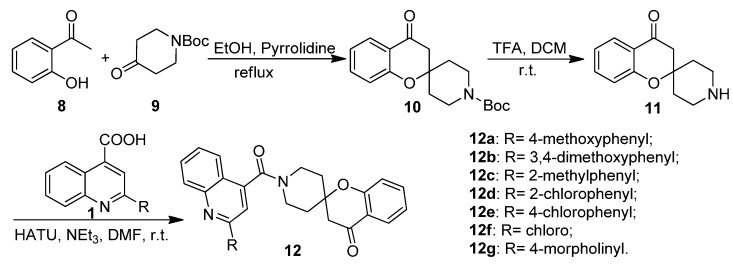
Synthesis of spirochromanones.

The synthetic routes for the novel target piperidinylpiperidine and spirochromanone derivatives are outlined in Scheme 1 and Scheme 2. As shown in Scheme 1, substituted 1-(quinoline-4-carbonyl)piperidin-4-ones **3** were obtained by the reaction of substituted quinoline-4-carboxylic acids **1** with piperidin-4-one hydrochloride (**2**) in the presence of HATU and triethylamine at room temperature. Derivatives of **3** then underwent reductive amination with (*R*)-ethyl piperidine-3-carboxylate hydrochloride (**4**) in the presence of sodium triacetoxyborohydride to afford substituted (*R*)-ethyl 1′-(quinoline-4-carbonyl)-[1,4′-bipiperidine]-3-carboxylates **5**. Derivatives of **5** further underwent hydrolysis and amidation to give the desired quinoline substituted piperidinylpiperidine derivatives **7**.

As shown in Scheme 2, the spirochromanones **12a**–**12g** were synthesized via a three-step procedure. The key intermediate *tert*-butyl 4-oxospiro[chroman-2,4′-piperidine]-1′-carboxylate (**10**) was prepared by the reaction of 1-(2-hydroxyphenyl)ethanone (**8**) with *N*-Boc-4-piperidinone (**9**) in a base catalyzed spirocyclization according to the reported procedure [7]. The Boc group on the intermediate was removed with trifluoroacetic acid (TFA) at room temperature, and the resulting spiro[chroman-2,4′-piperidin]-4-one (**11**) was further coupled with substituted quinoline-4-carboxylic acids **1** in the presence of HATU and triethylamine to afford the corresponding spirochromanone derivatives **12**.

The structures of all the synthesized piperidinylpiperidines **7** and spirochromanones **12** have been confirmed by NMR, IR and MS, and all the corresponding data can be found in the Experimental section.

### 2.2. Biological Activities

The *in vitro* inhibitory activities of compounds **7a**–**7i** and **12a**–**12g** against both ACC1 and ACC2 are listed in Table 1. Among the tested compounds, the selected potent compounds (ACC IC_50_ < 1000 nM) were further evaluated for their cytotoxicity against human embryonic lung fibroblast (HELF) cells using the 3-[4,5-dimethylthylthiazol-2-yl]-2,5-diphenyl-tetrazolium bromide (MTT) method [15]. For the convenience of structure-activity relationship analysis, compounds **7a**–**7i** and **12a**–**12g** were defined as piperidinylpiperidine derivatives and spirochromanone derivatives, respectively. As shown in Table 1, we can conclude that most of the compounds showed moderate to good activities against ACC1 and ACC2 and low toxic effects against normal human embryonic lung fibroblasts. For example, **7a**–**7g**, **12a**, **12b** and **12d** showed promising ACC2 inhibitory activities with IC_50_ values ranging from 172 nM to 940 nM and low cytotoxic activities (>100 μM). **7a**–**7e**, **7g**, **12a** and **12b** showed promising ACC1 inhibitory activity with IC_50_ values below 1000 nM. Moreover, **7a**–**7g** and **12d** displayed more potent ACC2 inhibitory activity compared to their anti-ACC1 activity and exhibited a relative selectivity. It is worth mentioning that the most active compound, the piperidinylpiperidine derivative **7a** displayed comparable inhibitory activity against ACC1/2 as the parent compound CP-640186. The octanol/water partition coefficients (miLogP) and drug-likeness model scores were also computed for all the compounds using the online molinspiration LogP calculation program and molsoft software, respectively. All the synthesized compounds showed moderate to good drug-likeness score ranging from 0.41 to 1.61, which is higher than CP-640186 (0.27). The logP values of most of the test compounds range from 3.18 to 5.0 within the acceptable criteria.

**Table 1:** LogP measurements, drug-likeness model scores, *in vitro* cytotoxicity assay and inhibitory activities of ACC1 and ACC2.

Comp.	ACC1 IC_50_ (nM) ^a^	ACC2 IC_50_ (nM) ^a^	TC_50_ (μM) ^a,b^	LogP ^d^	Drug-Likeness Model Score ^e^
**7a**	189 (±3)	172 (±7)	>100	3.48	1.14
**7b**	750 (±9)	360 (±10)	>100	5.03	1.61
**7c**	940 (±5)	205 (±9)	>100	4.38	1.49
**7d**	620 (±12)	294 (±3)	>100	3.48	1.38
**7e**	750 (±11)	382 (±15)	>100	4.03	1.4
**7f**	>1000	920 (±14)	N.T. ^c^	4.54	1.37
**7g**	860 (±3)	810 (±11)	>100	3.42	0.85
**7h**	>1000	>1000	N.T. ^c^	4.10	1.13
**7i**	>1000	>1000	N.T. ^c^	4.05	0.92
**12a**	600 (±7)	650 (±12)	>100	4.84	1.07
**12b**	760 (±11)	820 (±14)	>100	4.43	1.05
**12c**	>1000	>1000	N.T. ^c^	5.18	0.73
**12d**	>1000	940	N.T. ^c^	5.41	0.81
**12e**	>1000	>1000	N.T. ^c^	5.46	1.04
**12f**	>1000	>1000	N.T. ^c^	3.91	0.65
**12g**	>1000	>1000	N.T. ^c^	3.18	0.41
**CP-640186**	173 (±4)	185 (±5)	>100	3.59	0.27

^a^ Values are the arithmetic means of three experiments, standard deviation is given in parentheses; ^b^ TC_50_: Concentrations which reduce the cell viability by 50%; ^c^ N.T.: not test; ^d^ Calculated by [16]; ^e^ Calculated by [17].

### 2.3. Molecular Docking Study

The ACCase is a multienzyme complex composed of three function domains: biotin carboxylase domain (BC), biotin carboxyl carrier protein domain (BCCP) and carboxyltransferase domain (CT) [18]. The CT domain catalyzes the transfer of an activated carboxyl group to acetyl CoA to form malonyl CoA. The crystal structures of the CT domain in complex with CP-640186 [12], coenzyme A [19], and pinoxaden [20] have been reported, respectively. The cocrystal structures of CP-640186 reveal that the active site of the CT domain resides at the dimeric interface of the N and C domains. The binding pattern suggests that the anthracenyl moiety is sandwiched between helix α6 in the N domain and helix α6′ in the C domain near the dimer interface; the carbonyl oxygen atom adjacent to the anthracene is hydrogen bonded to Glu-2026 and the carbonyl oxygen atom adjacent to the morpholine is hydrogen bonded to Gly-1958.

**Figure 4:**
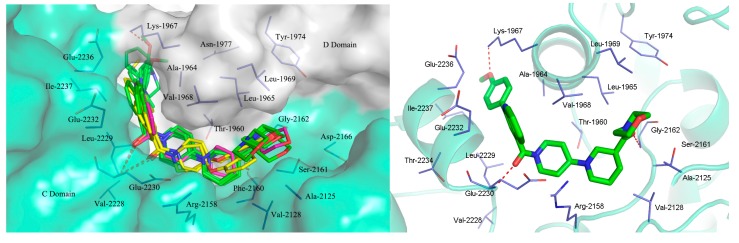
(**Left**) Overlay of the CT domain of hACC2 with the compounds of **7a**, **7g**, **7h**, **7i** (green), **12a**, **12e** (yellow) and CP-640186 (red), respectively. Red dashed lines denote the hydrogen bond. Molecular surface of the binding site is colored in white for the N domain and cyan for the C domain; (**Right**) Molecular docking model for compound **7a** (green and stick) with the active site of hACC2, highlighting the hydrogen bonds (red dashed lines) coordination between the oxygen atoms in **7a** and the amino acid residues Glu-2230, Gly-2162 and Lys-1967.

In order to investigate the interactions of these two series of compounds with ACC, a molecular docking study was performed by docking of several representative compounds into the active site of CT domain, using the Sybyl 7.1 program package (Tripos International, St. Louis, MO, USA). The three-dimensional structure of CT domain of human ACC was taken from the Protein Data Bank (PDB ID: 3FF6) [14]. The synthesized compounds were energy minimized and docked into the active site, which formed by residues Ala-1964, Lys-1967, Val-1968, Leu-1965, Gly-2162, Ser-2161, Phe-2160, Arg-2158, Glu-2230, Glu-2232, Glu-2236 and Ile-2237. The clustering of docked compounds **7a**, **7g**, **7h**, **7i**, **12a**, **12e** and CP-640186 is shown in Figure 4. These compounds exhibited similar conformations and binding modes as CP-640186. The best inhibitor **7a** with a higher docking score (8.01) in comparison to CP-640186 (7.18) and binds at the same site as the ligand. Two hydrogen bonds were observed between the carbonyl groups with residues Glu-2230 and Gly-2162 (Figure 4, right), respectively. The quinoline moiety is surrounded by the side chain of Ala-1964, Lys-1967, Val-1968, Gly-2233, Glu-2236 and Ile-2237. More importantly, the oxygen atom associated with 2-phenylquinoline moiety interacts with the residue Lys-1967 by an additional hydrogen bond. We speculate that the enhanced activity of **7a** is partly due to the additional hydrogen bond with Lys-1967.

## 3. Experimental Section

### 3.1. General Information

All solvents were dried according to standard methods before use. Melting points were measured on a Buchi B-545 melting point apparatus and are uncorrected. IR spectra were determined using KBr disks with a PE-983 infrared spectrometer and only major absorptions are listed. NMR spectra were recorded on a Mercury-Plus 400 spectrometer in CDCl_3_ using TMS as an internal reference. LC-MS data were obtained on an API 2000 liquid chromatography-tandem mass spectrometer. Unless otherwise noted, all materials were commercially available and were used directly without further purification.

#### 3.1.1. General Procedure for the Preparation of Substituted 1-(Quinoline-4-carbonyl)piperidin-4-ones **3**

To a solution of piperidin-4-one hydrochloride (**2**, 3.24 g, 24 mmol) and triethylamine (4.86 g, 48 mmol) in DMF (40 mL) was added substituted quinoline-4-carboxylic acid (**1**, 20 mmol) followed by HATU (9.12 g, 24 mmol). The reaction mixture was stirred at room temperature for 6 h and then poured into ice-cold water (50 mL). The precipitate was filtered and dried to give the corresponding substituted 1-(quinoline-4-carbonyl)piperidin-4-one **3**. This product was used directly in the next reaction step without further purification.

#### 3.1.2. General Procedure for the Preparation of Substituted (R)-Ethyl 1′-(Quinoline-4-carbonyl)-[1,4′-bipiperidine]-3-carboxylates **5**

A solution of (*R*)-ethyl piperidine-3-carboxylate hydrochloride (**4**, 2.32 g, 12 mmol) in 1,2-dichloroethane (20 mL) was treated with a substituted 1-(quinoline-4-carbonyl)piperidin-4-one **3** (10 mmol) followed by sodium triacetoxyborohydride (3.2 g, 15 mmol) and then acetic acid (4.5 mL). The suspension was stirred at room temperature for 2 h and then at 60 °C for 6 h. After cooling to room temperature, the reaction mixture was quenched with 1 N NaOH (5 mL), and stirred for 20 min. The suspension was extracted with CH_2_Cl_2_ (100 mL) and washed with water (50 mL). The organic layer was dried over Na_2_SO_4_, filtered, and concentrated to give a yellow residue which was purified by flash chromatography using EtOAc/petroleum ether as eluent.

#### 3.1.3. General Procedure for the Preparation of Substituted (R)-1′-(Quinoline-4-carbonyl)-[1,4′-bipiperidine]-3-carboxamides **7a**–**7i**

To a solution of substituted (*R*)-ethyl 1′-(quinoline-4-carbonyl)-[1,4′-bipiperidine]-3-carboxylate **5**, (5 mmol) in ethanol (15 mL) was added 1 N NaOH (20 mL). The reaction mixture was stirred at room temperature and monitored by TLC. Upon completion, the mixture was concentrated and acidified with 2 N HCl. The resulting solids were filtered, washed with H_2_O, and dried to provide (*R*)-1′-(quinoline-4-carbonyl)-[1,4′-bipiperidine]-3-carboxylic acid **6** which was used directly in the next step with no further purification. To a solution of the abovementioned acid **6** (5 mmol), and the amine (6 mmol) in DMF (5 mL) was added HATU (2.28 g, 6 mmol) followed by NEt_3_ (0.61 g, 6 mmol). The reaction mixture was stirred at room temperature for 6 h, then diluted with water (30 mL) and extracted with EtOAc (60 mL). The organics were dried with Na_2_SO_4_ and concentrated *in vacuo* to give crude compound **7**, which were purified by flash chromatography.

*(R)-(2-(4-Methoxyphenyl)quinolin-4-yl)(3-(morpholine-4-carbonyl)-[1,4′-bipiperidin]-1′-yl)methanone* (**7a**). Pale yellow solid, m.p. 94–96 °C. ^1^H-NMR δ (ppm): 8.17–8.11 (m, 3H), 7.85–7.71 (m, 3H), 7.51 (d, *J* = 8.0 Hz, 1H), 7.04 (t, *J* = 8.0 Hz, 2H), 4.99 (t, *J* = 10.0 Hz, 1H), 3.88 (s, 3H), 3.67–3.59 (m, 4H), 3.53–3.42 (m, 6H), 3.01–2.95 (m, 5H), 2.21–2.08 (m, 3H), 1.78–1.56 (m, 7H). IR *ν*: 3448.5, 1635.5, 1448.4, 1249.8, 842.8 cm^−1^. LC-MS calcd for C_32_H_39_N_4_O_4_ (*m*/*z*) [M + H]^+^ 543.30; found 543.28.

*(R)-N-Benzyl-1'-(2-(4-methoxyphenyl)quinoline-4-carbonyl)-N-methyl-[1,4'-bipiperidine]-3-carbox-amide* (**7b**). White solid, m.p. 90–92 °C. ^1^H-NMR δ (ppm): 8.26–8.13 (m, 3H), 7.89–7.80 (m, 3H), 7.75–7.70 (m, 2H), 7.64–7.47 (m, 4H), 7.06–7.01 (d, *J* = 8.0 Hz, 2H), 5.10 (t, *J* = 10.0 Hz, 1H), 4.20 (s, 2H), 3.87 (s, 3H), 3.68–3.60 (m, 4H), 3.53–3.38 (m, 4H), 3.08–2.90 (m, 3H), 2.03–1.93 (m, 5H), 1.78–1.56 (m, 4H). IR *ν*: 3442.7, 2936.5, 1631.7, 1448.4, 1109.0, 841.0 cm^−1^. LC-MS calcd for C_36_H_40_N_4_O_3_ (*m*/*z*) [M + H]^+^ 577.22; found 577.21.

*(R)-N,N-Diethyl-1'-(2-(4-methoxyphenyl)quinoline-4-carbonyl)-[1,4'-bipiperidine]-3-carboxamide* (**7c**). White solid, m.p. 101–102 °C. ^1^H-NMR δ (ppm): 8.16–8.11 (m, 3H), 7.81–7.70 (m, 3H), 7.51 (d, *J* = 8.0 Hz, 1H), 7.05 (t, *J* = 6.0 Hz, 2H), 5.00 (t, *J* = 10.0 Hz, 1H), 3.88 (s, 3H), 3.70–3.62 (m, 4H), 3.46–3.32 (m, 4H), 2.91–2.43 (m, 3H), 2.21–2.01 (m, 3H), 1.78–1.56 (m, 7H), 1.17 (t, *J* = 8.0 Hz, 3H), 1.09 (t, *J* = 8.0 Hz, 3H). IR *ν*: 3415.7, 3361.0, 1631.7, 1450.4, 1251.2, 842.8 cm^−1^. LC-MS calcd for C_32_H_40_N_4_O_3_ (*m*/*z*) [M + H]^+^ 529.32; found 529.30.

*(R)-(2-(4-Methoxyphenyl)quinolin-4-yl)(3-(4-methylpiperazine-1-carbonyl)-[1,4'-bipiperidin]-1'-yl)-methanone* (**7d**). White solid, m.p. 108–110 °C. ^1^H-NMR δ (ppm): 8.19–8.14 (m, 3H), 7.74–7.65 (m, 3H), 7.30 (d, *J* = 8.0 Hz, 1H), 7.23–6.99 (m, 2H), 4.97 (t, *J* = 10.0 Hz, 1H), 3.85 (s, 3H), 3.47–3.14 (m, 4H), 3.11–2.97 (m, 6H), 2.97–2.90 (m, 5H), 2.11–2.08 (m, 6H), 1.90–1.87 (m, 4H), 1.35–1.23 (m, 3H). IR *ν*: 3440.8, 1636.5, 1438.8, 1251.8, 769.5 cm^−1^. LC-MS calcd for C_33_H_41_N_5_O_3_ (*m*/*z*) [M + H]^+^ 556.31; found 556.29.

*(R)-(2-(4-Methoxyphenyl)quinolin-4-yl)(3-(pyrrolidine-1-carbonyl)-[1,4'-bipiperidin]-1'-yl)methanone* (**7e**). White solid, m.p. 127–129 °C. ^1^H-NMR δ (ppm): 8.19–8.11 (m, 3H), 7.79 (d, *J* = 8.0 Hz, 1H), 7.75–7.65 (m, 2H), 7.57–7.48 (m, 1H), 7.05 (d, *J* = 8.0 Hz, 2H), 4.98 (t, *J* = 10.0 Hz, 1H), 3.89 (s, 3H), 3.46–3.39 (m, 6H), 3.10–2.85 (m, 5H), 2.22–2.06 (bs, 2H), 1.96–1.90 (m, 4H), 1.85–1.71 (m, 8H); IR *ν*: 3415.7, 1623.9, 1452.3, 1251.7, 1026.1, 842.8 cm^−1^. LC-MS calcd for C_32_H_38_N_4_O_3_ (*m*/*z*) [M + H]^+^ 527.26; found 527.25.

*(R)-(2-(4-Methoxyphenyl)quinolin-4-yl)(3-(piperidine-1-carbonyl)-[1,4′-bipiperidin]-1′-yl)methanone* (**7f**). Pale yellow solid, m.p. 89–91 °C. ^1^H-NMR δ (ppm): 8.21–8.12 (m, 3H), 7.78 (d, *J* = 8.0 Hz, 1H), 7.76–7.64 (m, 2H), 7.60–7.51 (m, 1H), 7.08 (d, *J* = 8.0 Hz, 2H), 4.99 (t, *J* = 10.0 Hz, 1H), 3.92 (s, 3H), 3.52–3.43 (m, 6H), 3.12-2.84 (m, 5H), 2.31–2.24 (m, 2H), 2.02–1.92 (m, 4H), 1.80–1.64 (m, 10H) ; IR *ν*: 3426.8, 1632.6, 1460.9, 1258.6, 1034.5, 856.7 cm^−1^. LC-MS calcd for C_32_H_38_N_4_O_3_ (*m*/*z*) [M + H]^+^ 541.42; found 541.42.

*(R)-(3-(Morpholino-4-carbonyl)-[1,4'-bipiperidin]-1'-yl)(2-phenylquinolin-4-yl)methanone* (**7g**). White solid, m.p. 92–93 °C. ^1^H-NMR δ (ppm): 8.24 (d, *J* = 8.8 Hz, 2H), 7.91–7.80 (m, 3H), 7.67–7.63 (m, 1H), 7.55–7.52 (m, 2H), 7.39–7.33 (m, 2H), 4.97 (t, *J* = 10.0 Hz, 1H), 3.70–3.35 (m, 4H), 3.49–3.40 (m, 6H), 3.20–2.92 (m, 5H), 2.20–2.13 (m, 3H), 1.80–1.64 (m, 7H). IR *ν*: 3418.5, 1641.2, 1476.3, 1260.7, 1042.9, 867.6 cm^−1^. LC-MS calcd for C_31_H_36_N_4_O_3_ (*m*/*z*) [M + H]^+^ 513.29; found 513.31.

*(R)-(2-(4-Chlorophenyl)quinolin-4-yl)(3-(morpholine-4-carbonyl)-[1,4'-bipiperidin]-1'-yl)methanone* (**7h**). Pale yellow solid, m.p. 87–89 °C. ^1^H-NMR δ (ppm): 8.28–8.21 (m, 3H), 7.84 (d, *J* = 8.0 Hz, 1H), 7.70–7.65 (m, 2H), 7.65–7.58 (m, 1H), 7.36 (d, *J* = 8.0 Hz, 2H), 4.98 (t, *J* = 10.0 Hz, 1H), 3.76–3.48 (m, 4H), 3.50–3.42 (m, 6H), 3.25–2.96 (m, 5H), 2.24–2.16 (m, 3H), 1.87–1.66 (m, 7H). IR *ν*: 3420.6, 1646.8, 1464.5, 1270.6, 1038.4, 853.4 cm^−1^. LC-MS calcd for C_31_H_36_ClN_4_O_3_ (*m*/*z*) [M + H]^+^ 548.25; found 547.26.

*(R)-(2-(2-Chlorophenyl)quinolin-4-yl)(3-(morpholine-4-carbonyl)-[1,4'-bipiperidin]-1'-yl)methanone* (**7i**). White solid, m.p. 93–95 °C. ^1^H-NMR δ (ppm): 8.24–8.16 (m, 3H), 7.86–7.70 (m, 3H), 7.64–7.56 (m, 1H), 7.42–7.38 (m, 2H), 5.00 (t, *J* = 10.0 Hz, 1H), 3.78–3.48 (m, 4H), 3.52–3.43 (m, 6H), 3.42–3.12 (m, 5H), 2.30–2.21 (m, 3H), 1.92–1.72 (m, 7H); IR *ν*: 3452.6, 1642.8, 1426.9, 1247.5, 780.4 cm^−1^. LC-MS calcd for C_31_H_35_ClN_4_O_3_ (*m*/*z*) [M + H]^+^ 547.25; found 547.26.

#### 3.1.4. General Procedure for the Preparation of *Tert*-butyl 4-oxospiro[chroman-2,4' piperidine]-1'-carboxylate (**10**)

The synthesis was performed according to the reported method with some modifications [7]. Generally, a solution of 1-(2-hydroxyphenyl)ethanone (**8**, 4.08 g, 30 mmol) in ethanol (80 mL) was added pyrrolidine (2.04 g, 30 mmol). The solution was stirred for 2 h at room temperature and then, the reaction mixture was added *tert*-butyl 4-oxopiperidine-1-carboxylate (**9**, 5.97 g, 30 mmol) and stirred at room temperature for 10 h. The mixture was then concentrated *in vacuo* to give crude product which was purified via flash chromatography to afford *tert*-butyl 4-oxospiro[chroman-2,4′-piperidine]-1′-carboxylate **10** (6.84 g, 72% yield) as a white solid.

#### 3.1.5. General Procedure for the Preparation of Substituted 1′-(Quinoline-4-carbonyl)spiro[chroman-2,4′-piperidin]-4-ones **12a**–**12g**

To a solution of *tert*-butyl 4-oxospiro[chroman-2,4′-piperidine]-1′-carboxylate (1.6 g, 5 mmol) in dichloromethane (20 mL) was added trifluoroacetic acid (2.5 mL). The reaction mixture was stirred at room temperature for 2 h and the solvent was removed under reduced pressure to give crude spiro[chroman-2,4′-piperidin]-4-one **11**. The resulting solid was used directly for the next step reaction without further purification. A mixture of substituted quinoline-4-carboxylic acid (**1**, 5 mmol), NEt_3_ (0.61 g, 6 mmol) and HATU (1.9 g, 5 mmol) in DMF (10 mL) was stirred at room temperature for 0.5 h, followed by the abovementioned spiro[chroman-2,4′-piperidin]-4-one **11**. The reaction mixture was stirred overnight at room temperature after which time it was diluted with EtOAc (100 mL) and washed with water (60 mL × 2). The organic extract was dried over Na_2_SO_4_, filtered and concentrated under reduced pressure. The crude material was purified by chromatography to afford substituted 1′-(quinoline-4-carbonyl)spiro[chroman-2,4′-piperidin]-4-ones **12**.

*1'-(2-(4-Methoxyphenyl)quinoline-4-carbonyl)spiro[chroman-2,4'-piperidin]-4-one* (**12a**). White solid, m.p. 113–114 °C. ^1^H-NMR δ (ppm): 8.18–8.12 (m, 3H), 7.86 (d, *J* = 4.0 Hz, 1H), 7.80–7.71 (m, 3H), 7.55 (t, *J* = 10.0 Hz, 1H), 7.50–7.48 (m, 1H), 7.06–6.99 (m, 4H), 3.89 (s, 3H), 3.51–3.39 (m, 2H), 3.27 (m, 2H), 2.76 (s, 2H), 1.98–1.80 (m, 2H), 1.57–1.46 (m, 2H). IR *ν*: 1689.5, 1637.5, 1606.6, 1461.9, 1249.8, 763.76 cm^−1^. LC-MS calcd for C_30_H_26_N_2_O_4_ (*m*/*z*) [M + H]^+^ 479.19; found 479.18.

*1'-(2-(3,4-Dimethoxyphenyl)quinoline-4-carbonyl)spiro[chroman-2,4'-piperidin]-4-one* (**12b**). White solid, m.p. 121–123 °C. ^1^H-NMR δ (ppm): 8.20 (d, *J* = 8.0 Hz, 1H), 7.87 (d, *J* = 8.0 Hz, 2H), 7.81–7.77 (m, 3H), 7.68 (d, *J* = 8.0 Hz, 1H), 7.59–7.50 (m, 2H), 7.02 (q, *J* = 6.7 Hz, 3H), 4.77 (t, *J* = 12.0 Hz, 1H), 4.08 (s, 3H), 3.98 (s, 3H), 3.46 (t, *J* = 10.0 Hz, 2H), 3.28 (m, 2H), 2.73 (s, 2H), 1.98–1.83 (m, 2H), 1.60–1.48 (m, 2H). IR *ν*: 2929.7, 1637.5, 1461.9, 1261.4, 812.0, 765.7 cm^−1^. LC-MS calcd for C_31_H_28_N_2_O_5_ (*m*/*z*) [M + H]^+^ 509.22; found 509.23.

*1'-(2-(o-Tolyl)quinoline-4-carbonyl)spiro[chroman-2,4'-piperidin]-4-one* (**12c**). White solid, m.p. 126–127 °C. ^1^H-NMR δ (ppm): 8.19 (d, *J* = 8.0 Hz, 1H), 7.87–7.78 (m, 3H), 7.62 (t, *J* = 8.0 Hz, 1H), 7.52–7.45 (m, 3H), 7.37–7.32 (m, 3H), 7.01–6.99 (m, 2H), 3.53–3.40 (m, 2H), 2.75 (s, 2H), 2.43 (s, 3H), 2.32–2.26 (m, 2H), 1.97–1.82 (m, 2H), 1.60–1.46 (m, 2H). IR *ν*: 2923.9, 1697.2, 1635.5, 1483.9, 1228.6, 761.8 cm^−1^. LC-MS calcd for C_30_H_26_N_2_O_3_ (*m*/*z*) [M + H]^+^ 463.20; found 463.20.

*1'-(2-(2-Chlorophenyl)quinoline-4-carbonyl)spiro[chroman-2,4'-piperidin]-4-one* (**12d**). White solid, m.p. 130–132 °C. ^1^H-NMR δ (ppm): 8.21 (d, *J* = 8.0 Hz, 1H), 7.87 (d, *J* = 8.0 Hz, 2H), 7.81–7.77 (m, 1H), 7.71 (d, *J* = 8.0 Hz, 1H), 7.68–7.62 (m, 2H), 7.52–7.50 (m, 2H), 7.45–7.39 (m, 2H), 7.02 (t, *J* = 8.0 Hz, 2H), 3.56–3.49 (m, 2H), 3.48–3.40 (m, 2H), 2.77 (s, 2H), 1.99–1.83 (m, 2H), 1.69–1.62 (m, 2H). IR *ν*: 3438.8, 1693.4, 1639.4, 1463.9, 1226.6, 761.8 cm^−1^. LC-MS calcd for C_29_H_23_ClN_2_O_3_ (*m*/*z*) [M + H]^+^ 483.15; found 483.14.

*1'-(2-(4-Chlorophenyl)quinoline-4-carbonyl)spiro[chroman-2,4'-piperidin]-4-one* (**12e**). White solid, 128–130 °C. ^1^H-NMR δ (ppm): 8.21 (d, *J* = 8.0 Hz, 1H), 8.13 (d, *J* = 8.0 Hz, 2H), 7.88 (d, *J* = 8.0 Hz, 1H), 7.83–7.77 (m, 3H), 7.62 (t, *J* = 8.0 Hz, 1H), 7.54–7.51 (m, 3H), 7.03 (q, *J* = 6.7 Hz, 2H), 3.53–3.41 (m, 2H), 3.31–3.24 (m, 2H), 2.78 (s, 2H), 2.06–1.83 (m, 2H), 1.62–1.46 (m, 2H). IR *ν*: 2923.8, 1648.5, 1461.9, 1226.6, 835.1, 763.8 cm^−1^. LC-MS calcd for C_29_H_23_ClN_2_O_3_ (*m*/*z*) [M + H]^+^ 483.15; found; 483.14 [M + H]^+^.

*1'-(2-Chloroquinoline-4-carbonyl)spiro[chroman-2,4'-piperidin]-4-one* (**12f**). Pale yellow solid, m.p. 121–123 °C. ^1^H-NMR δ (ppm): 8.09 (d, *J* = 8.0 Hz, 1H), 7.88 (d, *J* = 8.0 Hz, 1H), 7.83–7.79 (m, 2H), 7.66–7.62 (m, 1H), 7.55–7.51 (m, 1H), 7.36–7.33 (m, 1H), 7.07–7.02 (m, 2H), 3.56–3.43 (m, 2H), 3.23–3.21 (m, 2H), 2.79 (s, 2H), 2.10–1.96 (m, 2H), 1.65–1.47 (m, 2H). IR *ν*: 3436.9, 3064.7, 1639.4, 1461.9, 1228.6, 758.0 cm^−1^. LC-MS calcd for C_23_H1_9_ClN_2_O_3_ (*m*/*z*) [M + H]^+^ 407.12; found; 407.12 [M + H]^+^.

*1'-(2-Morpholinoquinoline-4-carbonyl)spiro[chroman-2,4'-piperidin]-4-one* (**12g**). White solid, m.p. 138–140 °C. ^1^H-NMR δ (ppm): 8.11 (d, *J* = 8.0 Hz, 1H), 7.75 (d, *J* = 8.0 Hz, 1H), 7.62–7.50 (m, 3H), 7.32–7.30 (m, 1H), 7.06–6.99 (m, 2H), 6.93–6.88 (m, 1H), 3.88–3.84 (m, 4H), 3.77-3.68 (m, 4H), 3.48–3.44 (m, 2H), 3.29–3.23 (m, 2H), 2.78 (s, 2H), 1.94–1.81 (m, 2H), 1.57–1.47 (m, 2H). IR *ν*: 3417.6, 3381.0, 1604.7, 1444.6, 1116.71, 761.8 cm^−1^. LC-MS calcd for C_27_H_27_N_3_O_4_ (*m*/*z*) [M + H]^+^ 458.20; found; 458.20.

### 3.2. Molecular Docking

The molecular docking was performed and analyzed with the Sybyl 7.1 program package [21]. The X-ray structure of CT domain bounded with CP-640186 was taken from the Protein Data Bank (PDB ID: 3FF6). All water molecules and ligands were removed from the original enzyme structure. For docked ligands, non-polar hydrogens were added and Gasteiger-Huckl charges were assigned. To validate our docking procedure, the co-crystallized ligand was redocked into the active site of the hACC2 and the reasonable RMSD value of 1.9620 Å was obtained. Dock scores were evaluated by Consensus Score (CScore), which integrates a number of popular scoring functions for ranking the affinity of ligands bound to the active site of a receptor.

### 3.3. ACC1 and ACC2 in Vitro Assay

To investigate the biological activities of the synthesized compounds, the *in vitro* inhibitory activities of these compounds against both rat ACC1 and ACC2 were evaluated according to the previous experimental method with some changes [8]. The ACC1 and ACC2 enzymes were supplied by Huawei Pharmaceutical Co. Ltd (Shanghai, China). The compound CP-640186 was supplied by Selleck Chemicals Co. Ltd. (Houston, TX, USA) Briefly, the test compounds were diluted in 10% DMSO at 10 μM. Then, the solution was diluted with buffer solution until it reached the required concentration (1 nM to 10 μM) and 5μL of the dilution was added to a 50 μL reaction mixture containing 30 mM HEPES, pH 7.4, 2 mM MgCl_2_, 2 mM sodium citrate, 1 mM DTT, 25 μM ATP, 16 mM NaHCO_3_ and 15 μM acetyl-CoA. All of the enzymatic reactions were conducted at 30 °C for 60 min. The assay was performed using Kinase-Glo Plus luminescence kinase assay kit (Beijing, China), which measures kinase activity by quantifying the amount of ATP remaining in solution following a kinase reaction. The luminescent signal from the assay is correlated with the amount of ATP present and is correlated with the amount of kinase activity. The same concentrations of DMSO were tested in parallel and used as background control. The inhibition percentage was expressed as the average value from three independent experiments. CP-640186, an ACC1/ACC2 dual inhibitor developed by Pfizer, was used as a control.

### 3.4. In Vitro Cytotoxic Activities

In the cytotoxicity experiment, tetrazolium-based colorimetric assay (MTT test) was used to measure the cytotoxic effect of these selected potent compounds on normal cells [15,22]. Human embryonic lung fibroblast cell line (HELF, WI-38) was purchased from the Cell Resource Center, Chinese Academy of Medical Sciences (Beijing, China). Briefly, cells (5000 per well) were collected and seeded in 96-well plates. After incubation for 24 h, cells were exposed to fresh medium containing various concentrations of test compound at 37 °C. After incubation for 48 h, 20 μL of MTT tetrazolium salt dissolved in phosphate buffered saline (PBS) at a concentration of 5 mg/mL was added to each well and incubated in a CO_2_ incubator for 4 h. The medium was then aspirated from each well and 150 μL of DMSO was added to dissolve formazan crystals. The absorbance of each well was measured by ELx808 Microplate Reader (BioTek, Winooski, VT, USA) at a test wavelength of 490 nm. TC50, the drug concentration which reduces the cell viability by 50% was calculated by the Logit method.

## 4. Conclusions

In conclusion, we have designed and synthesized two series of novel ACC inhibitors bearing a quinoline moiety. The molecular docking study revealed that compounds from these two series possessed similar conformations and binding modes as CP-640186 in the CT domain of human ACC. The preliminary *in vitro* assay indicated that most of the synthesized compounds possessed considerable ACC1/2 inhibitory activities. Moreover, compound **7a** displayed comparable inhibitory activity against ACC2 as the parent compound CP-640186, which may partly due to the additional hydrogen bond with the side chain amide of Lys-1967. Further studies in animal models are in progress in our laboratory.
